# The Palliative Care Movement in India: Another Freedom Struggle or a Silent Revolution?

**DOI:** 10.4103/0973-1075.53495

**Published:** 2009

**Authors:** Cherian Koshy

**Affiliations:** Department of Palliative Care, Regional Cancer Centre, Trivandrum, India

**Keywords:** Palliative Care, Opioid availability, WHO Ladder

## Abstract

The message of palliative care in India has become a movement in several parts of India in a short span of time. The past two decades have seen palpable changes in the mindset of health care providers, and policy makers with respect to the urgency in providing palliative care. With a population of over a billion spread over a vast geo-political mosaic, the reach and reliability of palliative care programmes may appear staggering and insurmountable. Nonetheless we have reasons to be proud in that we have overcome several hurdles and is presently in a ‘consolidation mode’. It is only a matter of time before the *‘aam admi’* has access to good palliative care. Easing narcotic licensing procedures, creation of standard operating procedures for morphine availability and the passing of the ‘Palliative Care Policy’ by the Government of Kerala are commendable milestones. We are today having more of ‘silver linings’ and less of ‘dark clouds’.

‘We make a living by what we get; we make a ‘Life’ by what we give’

*–Sir Winston Churchill*

## INTRODUCTION

From obscurity to *quasi-* priority, we have come a long way, since the mid 1980s concerning palliative care in India. The ‘message which became a movement’ has opened transnational corridors and bridged cross-cultural barriers. From obsolescence, it has become an omnipresent jargon at least in our medical lexicon. In many Indian States, it is a household word in their respective mother tongues. Obstacles became opportunities. From a haunting past we are at the crossroads of a wanting present and daunting future. Like the transition from ‘stone age to space age’ we in India have in a short span of time achieved in setting trends and bench marks, creating, models, doing research and creating trainers and training programmes in palliative care. We even boast of an ‘Institute of Palliative Medicine in India’. ‘Compassion, Commonsense and Cleverness’ the trinity of virtues, we have *ad infinitum* in our motherland. Perhaps we need not major on cleverness and occasionally common sense becomes an ‘uncommon sense’ forming thus a major barrier in palliative care delivery.

## WHAT IS PAIN?

From the ‘shortest yet complete’ definition of pain by Sir Charles Sherrington as ‘the psychical and physical adjunct to an imperative protective stimulus’ in 1906,[1] we have succeeded in giving pain its ‘pride of place’ through various definitions and making it the fifth vital sign and ‘distress’ the sixth. From pain as a sensation, we emphasized it as a perception, and finally as ‘what the patient says hurts’ to which I may add, ‘pain is whatever the family also says hurts’, as ‘financial pain’ and psychosocial issues are important components in the Indian scenario. Understanding the ethos of ‘Total Pain’ we are succeeding in mustering support from volunteers, well wishers, and have come to the crucial conclusion that most of the problems of advanced diseases are ‘non-medical’ in nature, in that the community has a role to play.[2] Thus the ‘third sector’ or ‘civil society’, a fellowship of individuals ‘arrived’ (and continue to do so) to an arena of ‘uncoerced collective action around shared interests’ with its *lakshman rekha* drawn between the other players – the State, Family or Commercial interests.[3] Over periods of time the integration has become seamless.

However, pain is a relevant acronym in the present Indian Scenario in that, it could stand to mean ‘Palliative Awareness India's Need’, ‘Palliative Accessibility India's Need’,’ Palliative Availability India's Need’, and last but not the least ‘Palliative Affordability India's Need’. Every hour more than 60 patients die in India from cancer and in pain. Naturally seeds for pain relief were sowed initially in the country by professionals who treated or confronted cancer, and they were at Varanasi, Mumbai, Ahmadabad, Bangalore, and Trivandrum in the 1980s.[4] Pain is the commonest symptom associated with cancer the prevalence of which is 30-50% among patients on active treatment for solid tumors and from 70 to 90% among those with advanced disease.[5] We need to specially underscore contributions of several others from within and beyond our national borders to Palliative Care development in India; whom we venerate. For fear of missing out on names, I shall not venture to do so.

## THE OPIOID IMBROGLIO

The NDPS act of 1985 was an ephemeral moratorium on opioid usage. Thus, in the process of rectifying a ‘tragedy’ a ‘malady’ took form and function! Under the former dispensation Institutions needed a ‘narcotic possession license’, a license specifying the quota that could be possessed during the period of license, a license if the supplier was in another state, an import license, an export license and lastly a license to transport the drug- ‘a hurdle pentagon.’ The road map to avail morphine was a maze with bumps and detour signposting, characteristic of ‘our system.’ The jinx was finally broken. Through the pioneering work of persistent Indian Palliative Care Physicians and the Pain and Policy Study Group at Madison Wisconsin collective wisdom prevailed and it yielded positive results.[6]

## RHETORIC AND REALITY

Every congregation in the first quarter of every year has an eclectic assembly of ‘concerned individuals’ and in such annual events of the Indian Association of Palliative Care, for the past decade and half we have had always sessions with Drugs Controllers, Excise Commissioners, beaurocrats, and politicians sharing the dais alongside senior members of the Indian Association of Palliative Care (IAPC). These have always been occasions to introspect and feel elated at ‘promises’ dished out *ad nauseum.* Still only less than three percent of Indians have access to Palliative Care Services of any kind. Of the 28 Indian States and 7 Union territories, only 13 have eased narcotic licensing procedures. Whereas there has been a Government order exempting Palliative Care Centers from the need for a ‘Drug License’ to purchase, stock and dispense oral morphine, the conditions laid down for the same are that it should be supplied for State Government approved Palliative Care Centers, with conditions to be satisfied by the dealer and institution coming under the State Drugs Controller's scanner. Interpretation of such eased procedures has been erratic in several states, and often times we have taken one-step forward and two steps backwards. The State of Tamil Nadu has a Standard Operating Manual (SOP) for use of ‘Morphine in terminal Cancer patients.’ The WHO model in Palliative Care is to juxtapose palliative care into the treatment trajectory from day one. The SOP which was released for Kerala in March 2009, has deleted the words ‘terminal and cancer’ and instead has ‘any hospital, hospice or other Institutions providing pain relief and Palliative Care’ as being eligible to apply for Registered Medical Institution (RMI) status, provided they satisfy the other conditions laid down for Minimum Mandatory Requirements (MMR)

## THE STEP LADDER AND ‘STEP STUMBLE’

The WHO step ladder pattern has been a landmark, a watershed, a milestone in the pain management timeline. ‘By the clock, by the ladder and by mouth’ is the expression in any teaching sessions on pain management. ‘Beyond the ladder,’ ‘alongside the ladder,’ and the ladder for other symptom states have sprung up in later years, all to add ‘life to years’ of the beleaguered patient. The irony is that if opioids are not available the step ladder does not become a stepping stone, but a stumbling block in that we are stuck at step II necessitating prescription of more expensive analgesics. We halt at step II and stop there. A patient with palliative care needs requiring opioids and supportive medicines need not spend more than Rs 500 per month.[7] Regional Cancer Centre, at Trivandrum, and Kidwai Cancer Centre at Bangalore has been making oral morphine solution since the 1990s, and the RCC at Trivandrum started making oral morphine capsules since May 2007 (5 and 10 mgs), in collaboration with the College of Pharmaceutical Sciences which is in the same campus. We found the break up of expenses for a 10 mg capsule to be as follows.

### Morphine capsule 10 mgs

**Table:** 

Raw materials (morphine sulphate)	Rs 0.56
Empty shell	Rs 0.06
Exepients	Rs 0.02
Plastic container	Rs 0.04
Packing materials	Rs 0.02
Total	Rs 0.70

Certainly, we can ‘Make a difference and lead by example.’

## PALLIATIVE PULSE

Strong and bounding in Kerala it is either feeble or not felt in most parts of the country. Besides a literate and politically conscious population, another important but less highlighted reason for the high coverage of Palliative Care services in Kerala is the geography. Kerala is a ‘vertical slice of mother earth’ with good road and rail coverage. The width of the State varies from 35 to 125 kilometers. Another milestone was the Palliative Care policy that the government of Kerala passed (GO. (P) No. 109/2008/HandFWD dated 15-4-2008). Kerala has always been a ‘distant thunder with a different drum beat,’ a state of mind in Palliative Care. The role of the National Rural Health Mission (NRHM) is commendable and cannot be underestimated. Its ‘*Arogya Keralam Project’* or ‘healthy Kerala’ for Palliative Care is really a marketable ‘brand wear.’ Broad band and connectivity has put India on the global map in IT and communications. The RCC at Trivandrum availed this and has regular dedicated Tele-conferencing in Palliative Care with its satellite hilly center at Adimali near Munnar (altitude >5000 feet) christened ‘H E L P for the Hills’. HELP to mean ‘Hear, Ease, Link, Palliate.’[8]

## DEMOCRACY IN PALLIATIVE LANDSCAPE

This is Palliative Care ‘of the people, by the people and for the people.’ The world's largest democracy had this to give to the rest of mankind, through community owned initiatives. It is an irony that over eighty percent of Palliative Care initiatives are implemented through community based organizations or through NGOs. The Neighborhood Network in Palliative Care (NNPC) caught the attention of the world in palliative care provision. They started including all categories of people with chronic illnesses and included social discards and even those with psychiatric illnesses abandoned by families. With approximately 100 patients in every *Panchayat* at any given point of time requiring palliative care, there are around one lakh needy patients in Kerala. Projected for the country it could be just under 60 lakhs.[9]

## ‘LIFE AFTER LIFE’ - EVOLVING PARADIGM

Bereft of theology this is perhaps addressing ‘spiritual issues’ in the highly secular fabric of *‘Bharath.’* To me it is good end of life care with spiritual focus, rehabilitating a patient who has gone through the trauma of cancer or taking care of children, spouse, or siblings, on bereavement, when *‘one life has passed away and other lives await to be taken care of.’* Palliative Care workers have a key role to play in providing ‘life after life.’ Addressing issues of disenfranchised grief, i.e. grief that cannot be ‘openly acknowledged, publicly mourned or socially supported’ is of paramount importance.[10] Such grief can also be relevant in the emotive component of grief in a Palliative Care giver.

## CONCLUSION

A specialty fondly referred to as ‘low tech and high touch,’ depends on caring compassionate hands and we have many! Winds of change are sweeping albeit silently and Palliative Care awareness and delivery is a matter of time before it reaches the farthest corner of India. Our objective has to be to ‘minimize formality and maximize humanity’ and help patients and families make the difficult transition from being ‘seriously ill and fighting death to being terminally ill and seeking peace,’ India was declared a free nation on August 15^th^ 1947, but were we really free? - No there were pockets of resistance at Goa and Hyderabad which the Indian army had to free.[11] Let the silent revolution continue and opportunity windows in Palliative Care delivery open. Let us not be complacent, IAPC can also mean the Indian Army of Palliative Caregivers and, let us set a S M A R T (Specific, Measurable, Attainable, Realistic, and Time framed) G O A L (Good, Objective, Ambitious, and Life-enhancing) to achieve a pain free India.

‘The woods are lovely dark and deep; but I have miles to go and promises to keep before I sleep’

*–Robert Frost*
